# Aberrant Changes in Cortical Complexity in Right-Onset Versus Left-Onset Parkinson’s Disease in Early-Stage

**DOI:** 10.3389/fnagi.2021.749606

**Published:** 2021-11-08

**Authors:** Lin Zhang, Qin Shen, Haiyan Liao, Junli Li, Tianyu Wang, Yuheng Zi, Fan Zhou, Chendie Song, Zhenni Mao, Min Wang, Sainan Cai, Changlian Tan

**Affiliations:** ^1^Department of Radiology, The Second Xiangya Hospital, Central South University, Changsha, China; ^2^Department of Radiology, Affiliated Hangzhou First People’s Hospital, Zhejiang University School of Medicine, Hangzhou, China

**Keywords:** Parkinson’s disease, surface-based morphometry, laterality, side-of-onset, cortical complexity

## Abstract

There is increasing evidence to show that motor symptom lateralization in Parkinson’s disease (PD) is linked to non-motor features, progression, and prognosis of the disease. However, few studies have reported the difference in cortical complexity between patients with left-onset of PD (LPD) and right-onset of PD (RPD). This study aimed to investigate the differences in the cortical complexity between early-stage LPD and RPD. High-resolution T1-weighted magnetic resonance images of the brain were acquired in 24 patients with LPD, 34 patients with RPD, and 37 age- and sex-matched healthy controls (HCs). Cortical complexity including gyrification index, fractal dimension (FD), and sulcal depth was analyzed using surface-based morphometry *via* CAT12/SPM12. Familywise error (FWE) peak-level correction at *p* < 0.05 was performed for significance testing. In patients with RPD, we found decreased mean FD and mean sulcal depth in the banks of the left superior temporal sulcus (STS) compared with LPD and HCs. The mean FD in the left superior temporal gyrus (STG) was decreased in RPD compared with HCs. However, in patients with LPD, we did not identify significantly abnormal cortical complex change compared with HCs. Moreover, we observed that the mean FD in STG was negatively correlated with the 17-item Hamilton Depression Scale (HAMD) among the three groups. Our findings support the specific influence of asymmetrical motor symptoms in cortical complexity in early-stage PD and reveal that the banks of left STS and left STG might play a crucial role in RPD.

## Introduction

The asymmetrical motor symptoms and signs found in patients with Parkinson’s disease (PD) commonly persist over the course of the disease (Barrett et al., 2011; Lee et al., 2015; Miller-Patterson et al., 2018), which may contribute to distinguishing PD from other atypical Parkinsonian syndromes (Postuma et al., 2015). Various findings suggest that the side of motor onset symptoms in PD might have important implications regarding the symptoms, progression, and prognosis. For instance, right-onset PD (RPD) seems to be associated with language- (Amick et al., 2006) and verbal memory- (Verreyt et al., 2011) related cognitive impairment and was a risk factor for developing impulsive compulsive behavior (Phillipps et al., 2020) and apathy (Harris et al., 2013), whereas, left-onset PD (LPD) typically performed worse in visuospatial tasks (Verreyt et al., 2011) and was found to endorse more sleep behavior disorders (Baumann et al., 2014) and hallucinations (Stavitsky et al., 2008). RPD is associated with worse treatment response (Hanna-Pladdy et al., 2015) and more severe complications (Bay et al., 2019) with levodopa treatment, as well as worse prognosis than LPD (Baumann et al., 2014). However, the mechanisms involved in PD asymmetry have not yet been elucidated. The plausible mechanisms include handedness (van der Hoorn et al., 2012) and susceptibilities of the left substantia nigra (Blesa et al., 2011; Prasad et al., 2018; Fiorenzato et al., 2021).

Structural MRI imaging studies have found differences in gray matter (GM) volume and cortical thickness between LPD and RPD. Lee et al. (2015) reported that the right middle frontal gyrus and precuneus have lateralized GM loss in LPD, which were related to visuospatial memory impairment. Kim et al. (2014) reported that motor-related areas of the contralateral hemisphere showed thinning in early-stage, non-demented, patients with right-handed LPD compared with healthy controls (HC).

Except for cortical thickness, other surface-based morphometry (SBM) indices such as gyrification index (GI), fractal dimension (FD), and sulcal depth can characterize cortical complexity. GI is defined as the ratio between the inner surface size and the outer surface size of a convex hull. FD is a scale-free morphometric measure, which may be more sensitive to characterize structural differences than GI (Madan and Kensinger, 2016; Chen et al., 2020). Recent studies have also shown altered FD in a variety of neuropsychiatric and neurological diseases such as Alzheimer’s disease (Nicastro et al., 2020), amyotrophic lateral sclerosis (Hedderich et al., 2020), and transient ischemic attack (Lv et al., 2021). Sulcal depth was defined as the distance toward an idealized smooth brain surface (Lohmann, 1998). However, to date, none of the previous studies have investigated differences of cortical surface complexity (i.e., GI, FD, and sulcal depth) between patients with respect to the side of motor onset symptoms. Therefore, we aimed to investigate the differences in GI, FD, and sulcal depth among patients with early-stage LPD and RPD and matched HC using SBM *via* CAT12/SPM12.

## Materials and Methods

### Participants

Patients with PD who were diagnosed by two experienced neurologists based on the Movement Disorder Society Clinical Diagnostic Criteria for Parkinson’s disease (Postuma et al., 2015) were enrolled. The inclusion criteria were as follows: (1) modified Hoehn-Yahr (H-Y) stage ≤ 1.5; (2) no obvious cognitive impairment assessed by the Mini-Mental State Examination (MMSE) score; (3) right-handedness; (4) no history of other psychiatric or neurological diseases; (5) “off” state; (6) duration of illness ≤ 5 years; and (7) age ≤ 70 years. Subjects were excluded if they (1) had other diseases and treatments that could potentially affect brain function, such as atypical parkinsonism, cerebral trauma, stroke, and other diseases of the neurological system; (2) had contraindications to MRI or were unable to cooperate with an MRI scan and clinical scales; or (3) had an MMSE score less than the corresponding education degree, *n* = 3. MMSE scores of >17 for illiterate subjects, >20 for 1–6 years of education, and >23 for 7 or more years of education, which were defined as normal MMSE scores. According to the side of motor onset, patients with PD were divided into two groups, namely, LPD (*n* = 24) and RPD (*n* = 34). Right-handed HCs (*n* = 37) matched for age, sex, and education were enrolled from the local community.

### Magnetic Resonance Imaging Acquisition

Magnetic resonance imaging was performed using a 3.0 T MRI scanner (MAGNETOM Skyra; Siemens Healthineers, Erlangen, Germany). High-resolution, T1-weighted images were acquired (sagittal slices: 176, repetition time (TR): 1,900 ms, echo time (TE): 2.01 ms, flip angle: 9°, field of view: 256 × 256 mm^2^, voxel size = 1 × 1 × 1 mm, slice thickness: 1.0 mm (no slice gap).

### Preprocessing

All images were processed and analyzed using the CAT12 toolbox^1^ implemented in SPM12 (Wellcome Trust Center for Neuroimaging, London, United Kingdom^2^) for Matlab2013b. For the processing and analysis steps, preset parameters in accordance with the CAT12 user manual^3^ were used. All images were smoothed using a Gaussian kernel with 20-mm full width at half maximum, including GI, FD, and sulcal depth. All subjects passed both the visual quality inspection and the CAT12 data quality checks. The weighted average (IQR) of all scans ranged between 82.32 and 86.55%, which corresponded to a quality grade B.

### Statistical Analysis

Statistical analysis of clinical information was performed using SPSS version 22.0 software (SPSS Inc., Chicago, IL, United States). We performed statistical analyses of imaging data *via* the CAT12/SPM12 statistical module applying one-way ANOVA to each of the morphometric measures, with age, sex, and levodopa equivalent daily dose (LEDD) as the covariates. The “Estimate” incorporated in CAT12 was used to estimate surface models according to the manual. Familywise error (FWE) peak-level correction at *p* < 0.05 was performed for significance testing. The Desikan-Killiany atlas (Desikan et al., 2006) was used to estimate mean surface parameters. *Post hoc* comparisons were performed using Bonferroni correction (*p* < 0.05/3 = 0.017). Correlations between clinical data and abnormal morphometric change were assessed using Spearman’s coefficient (*p* < 0.05/5 = 0.01, Bonferroni corrected).

## Results

### Descriptive Analysis

There was no difference in sex, age, education, and MMSE among the three study groups. Furthermore, disease duration, Unified Parkinson’s Disease Rating Scale (UPDRS), the Unified Parkinson’s Disease Rating Scale, part III motor examination total score (UPDRS-III), Modified H-Y stage, LEDD, and the 17-item Hamilton Depression Scale (HADM) were comparable between the RPD and LPD groups. Demographic and clinical data are summarized in Table 1.

**TABLE 1:** Demographic and clinical data of study groups.

*N* =	HC	LPD	RPD	*p* (HC vs. all PD)	*p* (LPD vs. RPD)
	=37	=24	=34		
Gender (male/female)	17/20	13/11	20/14	0.546	0.724
Age, years	54.72 ± 6.81	55.75 ± 8.08	55.00 ± 7.81	0.208	0.427
Age of onset, years	–	54.13 ± 8.19	53.94 ± 7.91	–	0.322
Education, years	7.82 ± 2.96	6.58 ± 3.49	8.39 ± 3.56	0.213	0.608
Disease duration, years	–	1.73 ± 1.15	1.25 ± 0.93	–	0.292
LEDD, mg/day	–	8.33 ± 40.82	13.24 ± 43.18	–	0.403
Modified H-Y stage	–	1.17 ± 0.24	1.13 ± 0.22	–	0.285
UPDRS	–	17.91 ± 8.57	17.73 ± 8.49	–	0.927
UPDRS-III	–	11.67 ± 6.79	10.76 ± 4.78	–	0.233
MMSE	26.70 ± 3.05	26.25 ± 2.69	27.21 ± 2.68	0.131	0.477
HAMD	2.32 ± 3.01	6.00 ± 3.68	5.74 ± 3.65	0.211	0.516

*All data are presented as means ± SD. HC, healthy controls; PD, Parkinson’s disease; LPD, left-onset Parkinson’s disease; RPD, right-onset Parkinson’s disease; LEDD, levodopa equivalent daily dose; H-Y, Hoehn and Yahr; UPDRS, Unified Parkinson’s Disease Rating Scale; UPDRS-III, the Unified Parkinson’s Disease Rating Scale, part III motor examination total score; MMSE, Mini-Mental State Examination; HADM, the 17-item Hamilton Depression Scale; –, Data not available.*

### Gyrification Analysis

No significant differences in GI were found among the RPD, LPD, and HC groups.

### Fractal Dimension Analysis

**FIGURE 1:**
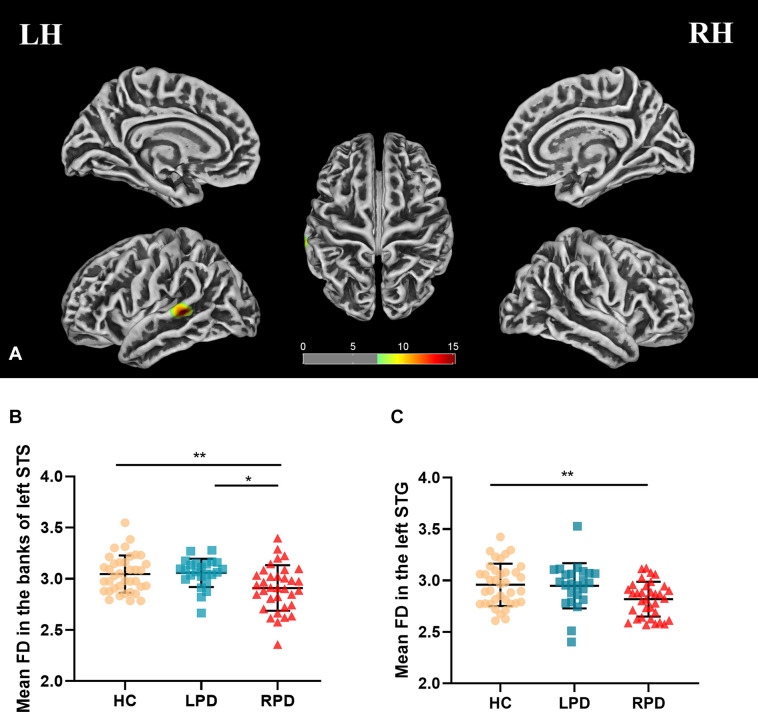
**(A)** Mean fractal dimension (FD) analysis of the group effect are highlighted [*p* < 0.05, familywise error (FWE) correction]. **(B,C)** Boxplots of the distribution of mean FD in the banks of the left superior temporal sulcus (STS) and left superior temporal gyrus (STG) among three groups (*post hoc p* < 0.017, Bonferroni corrected). *Post hoc* analyses revealed significantly decreased mean FD in the bank of left STS in patients with right-onset of Parkinson’s disease (RPD) compared with HC (**p = 0.009) and left-onset of PD (LPD) (*p = 0.012). Patients with RPD showed decreased mean FD in the left STG compared with HC (*p = 0.011).

Fractal dimension analysis revealed cluster-level significance (*F* = 12.9; *p* = 0.00001, FWE corrected) in a cluster comprising 456 vertices in the banks of the superior temporal sulcus (STS) and superior temporal gyrus (STG) of the left hemisphere. *Post hoc* analyses revealed significantly decreased mean FD in the bank of left STS in patients with RPD compared with HC (*p* = 0.009, Bonferroni corrected) and LPD (*p* = 0.012, Bonferroni corrected). When compared with HC, RPD showed decreased mean FD in the left STG (*p* = 0.011, Bonferroni corrected) (Figure 1). The cluster-level significant effects are summarized in Table 2.

**TABLE 2:** Overview of bilateral areas of cluster-level significant effects of cortical characteristics (FWE-corrected).

Hemisphere/cortical morphology	Overlap of atlas region	Cluster size	*p*-value	*F*	Peak MNI coordinates (x y z)
**Fractal dimension**					
LH	64% bankssts	999	0.00001	12.9	–57 –37 10
	36% superiortemporal				
**Sulcal depth**					
LH	100% bankssts	1111	0.00014	9.8	–57 –40 12
RH	96% postcentral	378	0.00001	13.8	22 –46 58
	4% precentral				

*Atlas labeling was performed according to the Desikan-Killiany atlas. LH, left hemisphere; RH, right hemisphere.*

### Sulcal Depth Analysis

**FIGURE 2:**
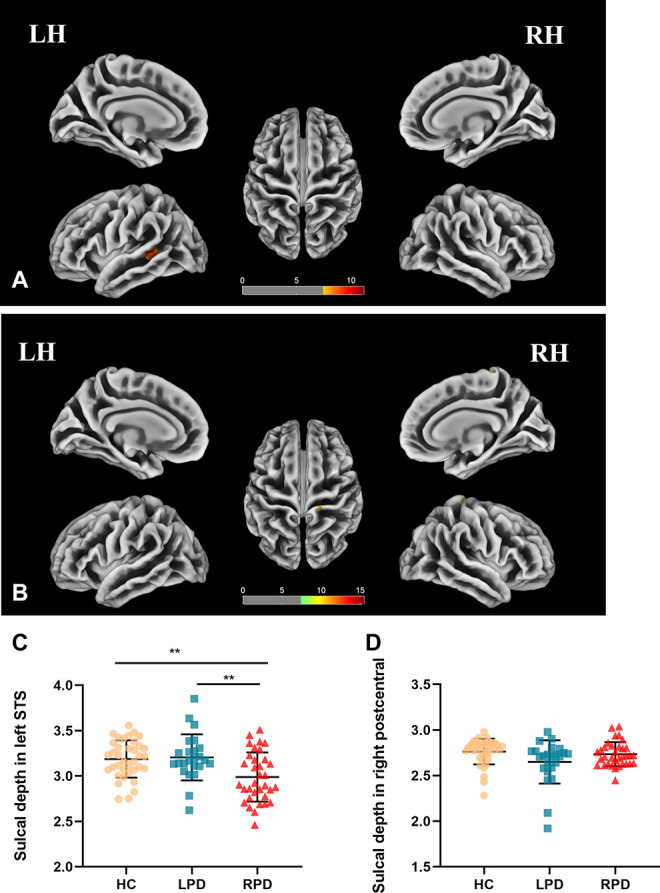
**(A,B)** Sulcal depth analysis of the group effect is highlighted (*p* < 0.05, FWE correction). **(C,D)** Boxplots of the distribution of sulcal depth in the banks of left STS and postcentral of the right hemisphere among the three groups (*post hoc p* < 0.017, Bonferroni corrected). *Post hoc* analyses revealed significantly decreased sulcal depth in the banks of left superior temporal of patients with RPD compared with HC (**p = 0.003) and LPD (**p = 0.004).

Cluster-level significant effects in the banks of left STG (*F* = 9.8; *p* = 0.00014, FWE corrected) and postcentral and precentral gyrus (*F* = 13.8; *p* = 0.00001, FWE corrected) of the right hemisphere (Table 2) were found. *Post hoc* analyses revealed significantly decreased sulcal depth in the banks of left STS of patients with RPD (Figure 2) compared with HC (*p* = 0.003, Bonferroni corrected) and LPD (*p* = 0.004, Bonferroni corrected). No decrease was found in the sulcal depth in the postcentral gyrus of the right hemisphere in patients with LPD compared with HC (*p* = 0.034 > 0.017) and RPD (*p* = 0.185 > 0.017).

### Correlational Analysis

**FIGURE 3:**
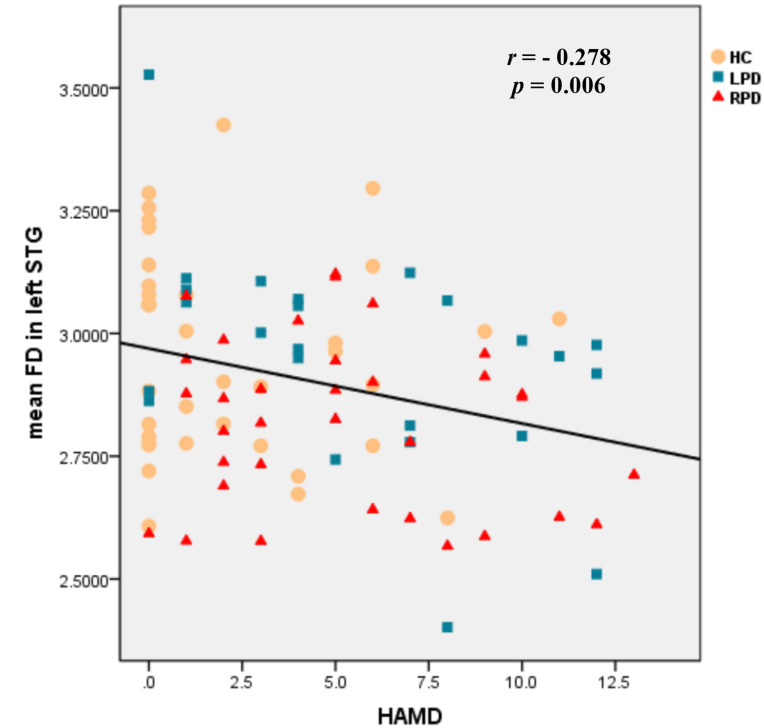
Scatter plots of the mean FD in the left STG negatively correlated with HAMD scores among the three groups (*r* = –0.278, *p* = 0.006, Bonferroni corrected).

We found that the mean FD in the left STG was negatively correlated with HAMD scores (*r* = –0.278, *p* = 0.006, Bonferroni corrected) among all three groups (Figure 3). For other regions listed in Table 2, no significant correlations were found between the mean cortical characteristics and the psychopathological data when we performed multiple comparison corrections.

## Discussion

This is the first study to investigate the differences in cortical surface complexity between patients with early-stage LPD and RPD by using SBM analysis. The mean FD and mean sulcal depth were lower in the banks of the left STS of patients with RPD than patients with LPD and HC. The mean FD in the left STG was decreased in RPD when compared with HC. However, in LPD, we did not observe any significantly abnormal cortical complex change compared with HC. In addition, the mean FD in left STG was negatively correlated with HAMD scores among the three groups.

Previous studies have shown that the banks of the STS were the core region, which accounted for verbal memory functions independent of the input modality (Ojemann et al., 2002) and engaged in supramodal language perception (Lindenberg and Scheef, 2007). The volume of this region, combined with the caudal portion of the anterior cingulate can also help differentiate between cognitively normal patients and those with mild cognitive impairment (Convit et al., 2000; DeVivo et al., 2019). In a cohort study, the denervation of the left hemisphere affected cognitive dysfunctions at onset and progression in right-handed PD (Fiorenzato et al., 2021). Recently, Guo et al. (2020) found that a high amyloid burden in the banks of the STS was predictive of memory decline over 4 years in Alzheimer’s disease (Park and Abner, 2020). Our study identified that RPD would develop abnormal structural changes in this area, which likely explains why RPD would perform worse in the language (Amick et al., 2006) and verbal memory tasks (Verreyt et al., 2011) than LPD.

The structure of the left STG is an important region for speech, language, and communication and plays a crucial role in the development of language abilities (Chen et al., 2004; Yagishita et al., 2008; Leff et al., 2009; Aeby et al., 2013; Vander Ghinst et al., 2016; Maruyama et al., 2018). Our results are in line with previous structural and functional studies that already pointed to the abnormalities of the left STG in PD (Wiesman et al., 2016; Suo et al., 2017; Gargouri et al., 2019; Yang et al., 2021). Similarly, a previous meta-analysis showed that PD with mild cognitive impairment (PD-MCI) had a robust GM decrease in the left STG (Qin et al., 2020). The GM volume of posterior STG was negatively linked to diadochokinetic (DDK) irregularity in PD with hypokinetic dysarthria (Klobusiakova et al., 2021). Moreover, when using low-frequency stimulation of STG, articulation in PD would be well-improved (Brabenec et al., 2019). Therefore, abnormal cortical complexity of the banks of left STS, along with the left STG in patients with RPD, suggests that those two regions might play a crucial role in RPD with cognitive impairment, which may serve as specific regions of interest for further investigations.

As a part of Wernicke’s region (Binder, 2017), the left STG and bank of the STS participate in the composition of the left fronto-temporo-parietal network (Kroczek et al., 2019), which is mainly related to language processing (Geranmayeh et al., 2016; Griffis et al., 2017; Kroczek et al., 2019) and working memory (Miró et al., 2020). PD-MCI is known to manifest language deficits (León-Cabrera et al., 2021; Letanneux et al., 2021) and decreased working memory (Caminiti et al., 2015), and in this part of patients were found that the network (Bayram et al., 2019; Jin Yoon et al., 2021) was damaged.

In contrast, previous studies (H-Y stage ≤ 2) did not show GM volume loss or cortical thinning in the left STG (Kim et al., 2014; Lee et al., 2015). The reasons for the inconsistency in results may be complicated by the different staging or disease durations of PD among these studies.

Furthermore, we also observed that mean FD in the left STG is negatively correlated with HAMD scores among the three groups, which was in line with previous studies. In fact, previous studies have shown a thinning left STG (Lebedeva et al., 2018; Wang et al., 2021) and altered functional connections between the left STG and anterior cingulate gyrus (Harada et al., 2018), as well as the left STG and the prefrontal cortex (Zhang et al., 2019) in depressive patients. Furthermore, the left STG was shown to likely be engaged in depression onset in patients with PD.

Finally, compared with HC, early-stage LPD showed no abnormal cortical complex, which has been explained by the greater vulnerability of the dominant hemisphere to PD-related dysfunction (Claassen et al., 2016). In line with previous publications, Pelizzari et al. (2020) showed that compared with HC, white matter integrity was found to be significantly altered in RPD but not in LPD in the early stage.

Our study has some limitations. First, the sample size was relatively small; therefore, the reliability of our findings should be conformed to a larger population. Second, although we found structural abnormalities in the left STG and the banks of left STS in patients with RPD, further correlation analysis could not be performed due to the lack of clinical evaluation data related to speech disorders. Third, some patients were not drug-naïve; although we assessed and controlled for current medication use (e.g., in an “off” state), the possible effects of medications cannot be entirely ruled out and may have been biased the results to some extent.

## Conclusion

Our results support the specific influence of asymmetrical motor symptoms in cortical complexity in early-stage PD. Further studies are required to assess the long-term evolution of asymmetry of motor onset symptoms and determine whether FD and sulcal depth represent a potential imaging marker for diagnostic and treatment strategies.

## Data Availability Statement

The original contributions presented in the study are included in the article/supplementary material, further inquiries can be directed to the corresponding author/s.

## Ethics Statement

The studies involving human participants were reviewed and approved by Medical Ethics Committee of the Second Xiangya Hospital, Central South University. The patients/participants provided their written informed consent to participate in this study.

## Author Contributions

LZ, JL, TW, YZ, ZM, FZ, CS, and MW: data collection. LZ, HL, QS, and SC: data analysis. LZ, QS, and JL: manuscript writing. CT: project development and manuscript revision. All authors: contributed to this study and approved the submitted version.

## Conflict of Interest

The authors declare that the research was conducted in the absence of any commercial or financial relationships that could be construed as a potential conflict of interest.

## Publisher’s Note

All claims expressed in this article are solely those of the authors and do not necessarily represent those of their affiliated organizations, or those of the publisher, the editors and the reviewers. Any product that may be evaluated in this article, or claim that may be made by its manufacturer, is not guaranteed or endorsed by the publisher.

## Footnotes

1
http://www.neuro.uni-jena.de/cat12/

2
http://www.fil.ion.ucl.ac.uk/spm/software/spm12/

3
http://www.neuro.uni-jena.de/cat12/CAT12-Manual.pdf

## References

[B1] Aeby A. De Tiège X. Creuzil M. David P. Balériaux D. Van Overmeire B. (2013). Language development at 2 years is correlated to brain microstructure in the left superior temporal gyrus at term equivalent age: a diffusion tensor imaging study. *Neuroimage* 78 145–151. 10.1016/j.neuroimage.2013.03.076 23583746

[B2] Amick M. M. Grace J. Chou K. L. (2006). Body side of motor symptom onset in Parkinson’s disease is associated with memory performance. *J. Int. Neuropsychol. Soc.* 12 736–740. 10.1017/s1355617706060875 16961953

[B3] Barrett M. J. Wylie S. A. Harrison M. B. Wooten G. F. (2011). Handedness and motor symptom asymmetry in Parkinson’s disease. *J. Neurol. Neurosurg. Psychiatry* 82 1122–1124. 20861062 10.1136/jnnp.2010.209783 PMC3729350

[B4] Baumann C. R. Held U. Valko P. O. Wienecke M. Waldvogel D. (2014). Body side and predominant motor features at the onset of Parkinson’s disease are linked to motor and nonmotor progression. *Mov. Disord.* 29 207–213. 10.1002/mds.25650 24105646

[B5] Bay A. A. Hart A. R. Michael Caudle W. Corcos D. M. Hackney M. E. (2019). The association between Parkinson’s disease symptom side-of-onset and performance on the MDS-UPDRS scale part IV: motor complications. *J. Neurol. Sci.* 396 262–265. 10.1016/j.jns.2018.12.002 30537631 PMC6324940

[B6] Bayram E. Bluett B. Zhuang X. Cordes D. Labelle D. R. Banks S. J. (2019). Neural correlates of distinct cognitive phenotypes in early Parkinson’s disease. *J. Neurol. Sci.* 399 22–29. 10.1016/j.jns.2019.02.013 30743154 PMC6436969

[B7] Binder J. R. (2017). Current Controversies on Wernicke’s Area and its Role in Language. *Curr. Neurol. Neurosci. Rep.* 17:58. 10.1007/s11910-017-0764-8 28656532

[B8] Blesa J. Juri C. García-Cabezas M. Á Adánez R. Sánchez-González M. Á Cavada C. (2011). Inter-hemispheric asymmetry of nigrostriatal dopaminergic lesion: a possible compensatory mechanism in Parkinson’s disease. *Front. Syst. Neurosci.* 5:92. 10.3389/fnsys.2011.00092 22287944 PMC3258666

[B9] Brabenec L. Klobusiakova P. Barton M. Mekyska J. Galaz Z. Zvoncak V. (2019). Non-invasive stimulation of the auditory feedback area for improved articulation in Parkinson’s disease. *Park. Relat. Disord.* 61 187–192. 10.1016/j.parkreldis.2018.10.011 30337204

[B10] Caminiti S. Siri C. Guidi L. Antonini A. Perani D. (2015). The neural correlates of spatial and object working memory in elderly and Parkinson’s disease subjects. *Behav. Neurol.* 2015:123636. 10.1155/2015/123636 25861157 PMC4378329

[B11] Chen H. H. Nicoletti M. A. Hatch J. P. Sassi R. B. Axelson D. Brambilla P. (2004). Abnormal left superior temporal gyrus volumes in children and adolescents with bipolar disorder: a magnetic resonance imaging study. *Neurosci. Lett.* 363 65–68. 15157998 10.1016/j.neulet.2004.03.042

[B12] Chen J.-H. Huang N.-X. Zou T.-X. Chen H.-J. (2020). Brain Cortical Complexity Alteration in Amyotrophic Lateral Sclerosis: a Preliminary Fractal Dimensionality Study. *Biomed Res. Int.* 2020:1521679. 10.1155/2020/1521679 32280675 PMC7115147

[B13] Claassen D. O. Mcdonell K. E. Donahue M. Rawal S. Wylie S. A. Neimat J. S. (2016). Cortical asymmetry in Parkinson’s disease: early susceptibility of the left hemisphere. *Brain Behav.* 6:e00573. 10.1002/brb3.573 28031997 PMC5167000

[B14] Convit A. De Asis J. De Leon M. Tarshish C. De Santi S. Rusinek H. (2000). Atrophy of the medial occipitotemporal, inferior, and middle temporal gyri in non-demented elderly predict decline to Alzheimer’s disease. *Neurobiol. Aging* 21 19–26. 10.1016/s0197-4580(99)00107-4 10794844

[B15] Desikan R. S. Ségonne F. Fischl B. Quinn B. T. Dickerson B. C. Blacker D. (2006). An automated labeling system for subdividing the human cerebral cortex on MRI scans into gyral based regions of interest. *NeuroImage* 31 968–980. 10.1016/j.neuroimage.2006.01.021 16530430

[B16] DeVivo R. Zajac L. Mian A. Cervantes-Arslanian A. Steinberg E. Alosco M. (2019). Differentiating Between Healthy Control Participants and Those with Mild Cognitive Impairment Using Volumetric MRI Data. *J. Int. Neuropsychol. Soc.* 25 800–810. 10.1017/s135561771900047x 31130145 PMC6995275

[B17] Fiorenzato E. Antonini A. Bisiacchi P. Weis L. Biundo R. (2021). Asymmetric Dopamine Transporter Loss Affects Cognitive and Motor Progression in Parkinson’s Disease. *Mov. Disord.* 36 2303–2313. 10.1002/mds.28682 34124799 PMC8596815

[B18] Gargouri F. Gallea C. Mongin M. Pyatigorskaya N. Valabregue R. Ewenczyk C. (2019). Multimodal magnetic resonance imaging investigation of basal forebrain damage and cognitive deficits in Parkinson’s disease. *Mov. Disord.* 34 516–525. 10.1002/mds.27561 30536444 PMC6590238

[B19] Geranmayeh F. Leech R. Wise R. J. S. (2016). Network dysfunction predicts speech production after left hemisphere stroke. *Neurology* 86 1296–1305. 10.1212/wnl.0000000000002537 26962070 PMC4826341

[B20] Griffis J. C. Nenert R. Allendorfer J. B. Szaflarski J. P. (2017). Linking left hemispheric tissue preservation to fMRI language task activation in chronic stroke patients. *Cortex* 96 1–18. 10.1016/j.cortex.2017.08.031 28961522 PMC5675757

[B21] Guo T. Landau S. Jagust W. (2020). Detecting earlier stages of amyloid deposition using PET in cognitively normal elderly adults. *Neurology* 94 e1512–e1524. 10.1212/WNL.0000000000009216 32188766 PMC7251521

[B22] Hanna-Pladdy B. Pahwa R. Lyons K. E. (2015). Paradoxical effect of dopamine medication on cognition in Parkinson’s disease: relationship to side of motor onset. *J. Int. Neuropsychol. Soc.* 21 259–270. 10.1017/S1355617715000181 25923830 PMC4493897

[B23] Harada K. Ikuta T. Nakashima M. Watanuki T. Hirotsu M. Matsubara T. (2018). Altered Connectivity of the Anterior Cingulate and the Posterior Superior Temporal Gyrus in a Longitudinal Study of Later-life Depression. *Front. Aging Neurosci.* 10:31. 10.3389/fnagi.2018.00031 29472854 PMC5809471

[B24] Harris E. Mcnamara P. Durso R. (2013). Apathy in patients with Parkinson disease as a function of side of onset. *J. Geriatr. Psychiatry Neurol.* 26 95–104. 10.1177/0891988713481267 23584852

[B25] Hedderich D. M. Bäuml J. G. Menegaux A. Avram M. Daamen M. Zimmer C. (2020). An analysis of MRI derived cortical complexity in premature-born adults: regional patterns, risk factors, and potential significance. *Neuroimage* 208:116438. 10.1016/j.neuroimage.2019.116438 31811902

[B26] Jin Yoon E. Ismail Z. Kathol I. Kibreab M. Hammer T. Lang S. (2021). Patterns of brain activity during a set-shifting task linked to mild behavioral impairment in Parkinson’s disease. *NeuroImage Clin.* 30:102590. 10.1016/j.nicl.2021.102590 33640685 PMC7907973

[B27] Kim J. S. Yang J. J. Lee J. M. Youn J. Kim J. M. Cho J. W. (2014). Topographic pattern of cortical thinning with consideration of motor laterality in Parkinson disease. *Park. Relat. Disord.* 20 1186–1190. 10.1016/j.parkreldis.2014.08.021 25231669

[B28] Klobusiakova P. Mekyska J. Brabenec L. Galaz Z. Zvoncak V. Mucha J. (2021). Articulatory network reorganization in Parkinson’s disease as assessed by multimodal MRI and acoustic measures. *Park. Relat. Disord.* 84 122–128. 10.1016/j.parkreldis.2021.02.012 33609963

[B29] Kroczek L. O. H. Gunter T. C. Rysop A. U. Friederici A. D. Hartwigsen G. (2019). Contributions of left frontal and temporal cortex to sentence comprehension: evidence from simultaneous TMS-EEG. *Cortex* 115 86–98. 10.1016/j.cortex.2019.01.010 30776735

[B30] Lebedeva A. Sundström A. Lindgren L. Stomby A. Aarsland D. Westman E. (2018). Longitudinal relationships among depressive symptoms, cortisol, and brain atrophy in the neocortex and the hippocampus. *Acta Psychiatr. Scand.* 137 491–502. 10.1111/acps.12860 29457245

[B31] Lee E. Y. Sen S. Eslinger P. J. Wagner D. Kong L. Lewis M. M. (2015). Side of motor onset is associated with hemisphere-specific memory decline and lateralized gray matter loss in Parkinson’s disease. *Park. Relat. Disord.* 21 465–470. 10.1016/j.parkreldis.2015.02.008 25749355 PMC4424064

[B32] Leff A. P. Schofield T. M. Crinion J. T. Seghier M. L. Grogan A. Green D. W. (2009). The left superior temporal gyrus is a shared substrate for auditory short-term memory and speech comprehension: evidence from 210 patients with stroke. *Brain* 132 3401–3410. 10.1093/brain/awp273 19892765 PMC2792373

[B33] León-Cabrera P. Pagonabarraga J. Morís J. Martínez-Horta S. Marín-Lahoz J. Horta-Barba A. (2021). Neural signatures of predictive language processing in Parkinson’s disease with and without mild cognitive impairment. *Cortex* 141 112–127. 10.1016/j.cortex.2021.03.032 34049254

[B34] Letanneux A. Velay J. Viallet F. Pinto S. (2021). Altered Inhibitory Mechanisms in Parkinson’s Disease: evidence From Lexical Decision and Simple Reaction Time Tasks. *Fronti. Hum. Neurosci.* 15:624026. 10.3389/fnhum.2021.624026 33981205 PMC8107209

[B35] Lindenberg R. Scheef L. (2007). Supramodal language comprehension: role of the left temporal lobe for listening and reading. *Neuropsychologia* 45 2407–2415. 10.1016/j.neuropsychologia.2007.02.008 17451759

[B36] Lohmann G. (1998). Extracting line representations of sulcal and gyral patterns in MR images of the human brain. *IEEE Trans. Med. Imaging* 17 1040–1048. 10.1109/42.746714 10048861

[B37] Lv Y. Wei W. Han X. Song Y. Han Y. Zhou C. (2021). Multiparametric and multilevel characterization of morphological alterations in patients with transient ischemic attack. *Hum. Brain Mapp*. 42 2045–2060. 10.1002/hbm.25344 33463862 PMC8046078

[B38] Madan C. R. Kensinger E. A. (2016). Cortical complexity as a measure of age-related brain atrophy. *NeuroImage* 134 617–629. 10.1016/j.neuroimage.2016.04.029 27103141 PMC4945358

[B39] Maruyama T. Takeuchi H. Taki Y. Motoki K. Jeong H. Kotozaki Y. (2018). Effects of Time-Compressed Speech Training on Multiple Functional and Structural Neural Mechanisms Involving the Left Superior Temporal Gyrus. *Neural Plast.* 2018:6574178. 10.1155/2018/6574178 29675038 PMC5838482

[B40] Miller-Patterson C. Buesa R. Mclaughlin N. Jones R. Akbar U. Friedman J. H. (2018). Motor asymmetry over time in Parkinson’s disease. *J. Neurol. Sci.* 393 14–17. 30096567 10.1016/j.jns.2018.08.001

[B41] Miró J. Ripollés P. Sierpowska J. Santurino M. Juncadella M. Falip M. (2020). Autobiographical memory in epileptic patients after temporal lobe resection or bitemporal hippocampal sclerosis. *Brain Imaging Behav.* 14 1074–1088. 10.1007/s11682-019-00113-8 31102166

[B42] Nicastro N. Malpetti M. Cope T. E. Bevan-Jones W. R. Mak E. Passamonti L. (2020). Cortical Complexity Analyses and Their Cognitive Correlate in Alzheimer’s Disease and Frontotemporal Dementia. *J. Alzheimers Dis.* 76 331–340. 10.3233/jad-200246 32444550 PMC7338220

[B43] Ojemann G. Schoenfield-Mcneill J. Corina D. (2002). Anatomic subdivisions in human temporal cortical neuronal activity related to recent verbal memory. *Nat. Neurosci.* 5 64–71. 10.1038/nn785 11753418

[B44] Park D. Abner E. (2020). Amyloid deposits in the banks (of the superior temporal sulcus) yield a high return about memory futures. *Neurology* 94 603–604. 10.1212/WNL.0000000000009213 32188764

[B45] Pelizzari L. Di Tella S. Laganà M. M. Bergsland N. Rossetto F. Nemni R. (2020). White matter alterations in early Parkinson’s disease: role of motor symptom lateralization. *Neurol. Sci.* 41 357–364. 10.1007/s10072-019-04084-y 31650438

[B46] Phillipps C. Longato N. Béreau M. Carrière N. Lagha-Boukbiza O. Mengin A. C. (2020). Is Motor Side Onset of Parkinson’s Disease a Risk Factor for Developing Impulsive-Compulsive Behavior? A Cross-Sectional Study. *Mov. Disord.* 35 1080–1081. 10.1002/mds.28053 32311121

[B47] Postuma R. B. Berg D. Stern M. Poewe W. Olanow C. W. Oertel W. (2015). MDS clinical diagnostic criteria for Parkinson’s disease. *Mov. Disord.* 30 1591–1601. 26474316 10.1002/mds.26424

[B48] Prasad S. Saini J. Yadav R. Pal P. K. (2018). Motor asymmetry and neuromelanin imaging: concordance in Parkinson’s disease. *Park. Relat. Disord.* 53 28–32. 10.1016/j.parkreldis.2018.04.022 29709506

[B49] Qin B. Yang M. X. Gao W. Zhang J. D. Zhao L. B. Qin H. X. (2020). Voxel-wise meta-analysis of structural changes in gray matter of Parkinson’s disease patients with mild cognitive impairment. *Braz. J. Med. Biol. Res.* 53:e9275. 10.1590/1414-431x20209275 32428131 PMC7266500

[B50] Stavitsky K. Mcnamara P. Durso R. Harris E. Auerbach S. Cronin-Golomb A. (2008). Hallucinations, dreaming, and frequent dozing in Parkinson disease: impact of right-hemisphere neural networks. *Cogn. Behav. Neurol.* 21 143–149. 10.1097/WNN.0b013e318185e698 18797256 PMC2630478

[B51] Suo X. Lei D. Li N. Cheng L. Chen F. Wang M. (2017). Functional Brain Connectome and Its Relation to Hoehn and Yahr Stage in Parkinson Disease. *Radiology* 285 904–913. 10.1148/radiol.2017162929 28873046

[B52] van der Hoorn A. Burger H. Leenders K. L. De Jong B. M. (2012). Handedness correlates with the dominant Parkinson side: a systematic review and meta-analysis. *Mov. Disord.* 27 206–210. 10.1002/mds.24007 21994149

[B53] Vander Ghinst M. Bourguignon M. Op De Beeck M. Wens V. Marty B. Hassid S. (2016). Left Superior Temporal Gyrus Is Coupled to Attended Speech in a Cocktail-Party Auditory Scene. *J. Neurosci.* 36 1596–1606. 10.1523/JNEUROSCI.1730-15.2016 26843641 PMC6601992

[B54] Verreyt N. Nys G. M. S. Santens P. Vingerhoets G. (2011). Cognitive differences between patients with left-sided and right-sided Parkinson’s disease. A review. *Neuropsychol. Rev.* 21 405–424. 10.1007/s11065-011-9182-x 21956794

[B55] Wang Y. Jiang P. Tang S. Lu L. Bu X. Zhang L. (2021). Left superior temporal sulcus morphometry mediates the impact of anxiety and depressive symptoms on sleep quality in healthy adults. *Soc. Cogn. Affect. Neurosci.* 16 492–501. 10.1093/scan/nsab012 33512508 PMC8095089

[B56] Wiesman A. I. Heinrichs-Graham E. Mcdermott T. J. Santamaria P. M. Gendelman H. E. Wilson T. W. (2016). Quiet connections: reduced fronto-temporal connectivity in nondemented Parkinson’s Disease during working memory encoding. *Hum. Brain Mapp.* 37 3224–3235. 10.1002/hbm.23237 27151624 PMC4980162

[B57] Yagishita S. Watanabe T. Asari T. Ito H. Kato M. Ikehira H. (2008). Role of left superior temporal gyrus during name recall process: an event-related fMRI study. *Neuroimage* 41 1142–1153. 10.1016/j.neuroimage.2008.03.008 18434201

[B58] Yang Q. Nanivadekar S. Taylor P. A. Dou Z. Lungu C. I. Horovitz S. G. (2021). Executive function network’s white matter alterations relate to Parkinson’s disease motor phenotype. *Neurosci. Lett.* 741:135486. 10.1016/j.neulet.2020.135486 33161103 PMC7750296

[B59] Zhang H. Qiu Y. Luo Y. Xu P. Li Z. Zhu W. (2019). The relationship of anxious and depressive symptoms in Parkinson’s disease with voxel-based neuroanatomical and functional connectivity measures. *J. Affect. Disord.* 245 580–588. 10.1016/j.jad.2018.10.364 30439681

